# Immune Niche Formation Reveals Mechanisms of Tumor Dormancy and Targeting Opportunities

**DOI:** 10.21203/rs.3.rs-8167536/v1

**Published:** 2026-02-02

**Authors:** Abdul Ahad, Feng Leng, Hiroshi Ichise, Edward Schrom, Jae Young So, Carter Sellner, Yang Gu, Wenjuan Wang, Celine Kieu, Woo Yong Park, Rachel Yang, Karen Wolcott, Ferenc Livak, Michael Kruhlak, Olga Aprelikova, Justin Gray, Vishal N. Kopardé, Yasuhiro Moriwaki, Ronald N. Germain, Li Yang

**Affiliations:** 1 Laboratory of Cancer Biology and Genetics, Center for Cancer Research, National Cancer Institute, National Institutes of Health, Bethesda, MD 20892; 2 Center for Advanced Tissue Imaging, Laboratory of Immune System Biology, National Institute of Allergy and Infectious Diseases, NIH, Bethesda, MD 20892; 3 Laboratory of Genome Integrity, Flow Cytometry Core, Center for Cancer Research, National Cancer Institute, National Institutes of Health, Bethesda, MD 20892; 4 Bioinformatics, CCBR (CCR Collaborative Bioinformatics Resource), National Cancer Institute, National Institutes of Health, Bethesda, MD 20892; 5 Department of Pharmacology, Keio University, Faculty of Pharmacy, Tokyo, Japan; 6 Lymphocyte Biology Section, Laboratory of Immune System Biology, National Institute of Allergy and Infectious Diseases, NIH, Bethesda, MD 20892

**Keywords:** Tumor dormancy, immune, niche, metastasis, relapse, TGF-β

## Abstract

Residual tumor cells can persist in a dormant state during clinical remissions that may last decades. The mechanisms leading to such growth control vs. eventual macroscopic metastases remain unclear. Here, we report abrogation of myeloid TGF-β RII resulted in an IFN-γ rich microenvironment. IFN-γ in turn elevated KLF4-mediated SLURP1 production in malignant cells, which is critical to their quiescent state through interruption of fibronectin-integrin pathways. The dormant tumor lesions were found in spatially localized immune niches rich in NK cells, cDCs, monocytes, and neutrophils, concomitant with tumor cell inactivation of NK cell immune surveillance through CD200-CD200R1. Our studies identify the IFN-γ-KLF4-SLURP1 and CD200-CD200R1 axes as critical molecular drivers in tumor dormancy regulated by immune-tumor crosstalk. Targeting the CD200-mediated dormant niche in combination with chemotherapy and immune check point blockade (ICB) significantly eradicated the dormant tumor cells. These insights provide mechanistic understanding of tumor dormancy and treatment options for ICB relapse.

## Introduction

Residual tumor cells can persist in a dormant state during extended periods of clinical remission^1–5^, with the ‘reawakening’ of dormant tumor cells after months to years often resulting in incurable metastatic outgrowth and patient mortality^1, 2, 6–8^. Such quiescence has been recognized as a key mechanism by which tumor cells can evade host immune detection, clearance, and therapeutic targeting^5, 9–13^ but progress in understanding the mechanisms governing tumor dormancy has been limited^5, 8, 14^.

Tumor dormancy can be characterized as cellular dormancy, that is, singular or a small cluster of quiescent cells without ongoing cell proliferation^15, 16^, or tumor mass dormancy, which involves micro-metastatic nodules that maintain an equilibrium between cell proliferation and intrinsic or immune-mediated death with no net increase in size over an extended period of time^1, 8, 17–21^. The quiescent state in cellular dormancy offers not only tumor cell protection from many conventional chemotherapies that rely on cell cycle progression to exert their cytotoxic effect but also promotes evasion of the host immune system^12, 22^. Several mechanisms regulating cellular dormancy have been identified, including those involving signaling pathways (e.g., TGF-β2, ERK1/2, and P-p38)^23, 24^, nuclear receptor NR2F1^25–27^, stemness factors^10, 28, 29^, and extracellular matrix mediators^30–35^. In addition, recent studies support the ability of dormant tumor cells to evade CD8^+^ T cell– and natural killer cell (NK cell)–mediated detection and clearance^12, 21, 29, 36–39^, with constraint of reactivated metastatic cells being analogous to control of latent / chronic viral infection with cytomegalovirus or herpes simplex virus. With these pathogens, episodic activation of latent virus is immediately targeted by the adaptive immune system to prevent overt viremia^40–42^ – in the case of cancer, such constant immunosurveillance and anti-tumor effector responses may prevent macroscopic tumor formation over an extended period, with eventual immune escape accounting for development of clinically relevant metastatic disease^19, 43^.

At present there is a lack of mechanistic insight into how disseminated tumor cells enter a reversible growth-arrest or quiescence state and what causal roles immune cells and molecular mediators play in keeping dormant cancer cells from being reactivated or developing into a clinically relevant tumor^44^. Additionally, it is unclear whether distinct immune niches, either microenvironments that are pre-organized by the host or ones created by disseminated tumor cells, actively facilitate tumor dormancy^4, 8^. One of the major challenges in filling these knowledge gaps is the lack of immune-competent mouse models that can effectively produce a long-term tumor dormancy phenotype *in vivo*, especially in the context of immune regulation^44, 45^.

Our studies over the last two decades have uncovered a key role for myeloid TGF-β signaling in promoting tumor immune evasion and metastatic outgrowth^46–49^. Here we use this model with a combination of advanced imaging, transcriptomics, and molecular studies to gain deeper insights into how modulation of myeloid cell innate immune state can prevent tumor outgrowth. We show that abrogation of myeloid TGF-β signaling induced an IFN-γ rich immune milieu, as expected and desired for immunotherapy including ICB. However, this IFN-γ rich immune microenvironment led to KLF4-SLURP1 upregulation that facilitates the tumor dormant state. The microdomains of dormant tumors contained accumulations of myeloid and lymphoid immune cells and this microdomain modulation is complemented by resistance of the tumor cells to NK cell-mediated killing via IFN-γ induced upregulation of CD200 and CD200R1 interactions. Together, these results reveal an unexpected duality of host anti-tumor immunity in promoting prolonged maintenance of viable metastatic tumor cells by facilitating entry into the dormant state in concert with increased expression of immune escape mechanisms. Further human correlative studies reveal a poor prognosis and early post-treatment relapse associated with tumor cell IFN-γ-KLF4-SLURP1 and CD200-CD200R1 gene signatures. Targeting the CD200-mediated dormant niche in preclinical mouse models significantly eradicated the dormant tumor cells, which opens avenues for prevention of metastatic relapse, especially those associated with ICB.

## Results

### Abrogation of myeloid-specific TGF-β signaling induces cellular tumor dormancy in multiple mouse models of breast cancer metastasis

**Figure 1.**
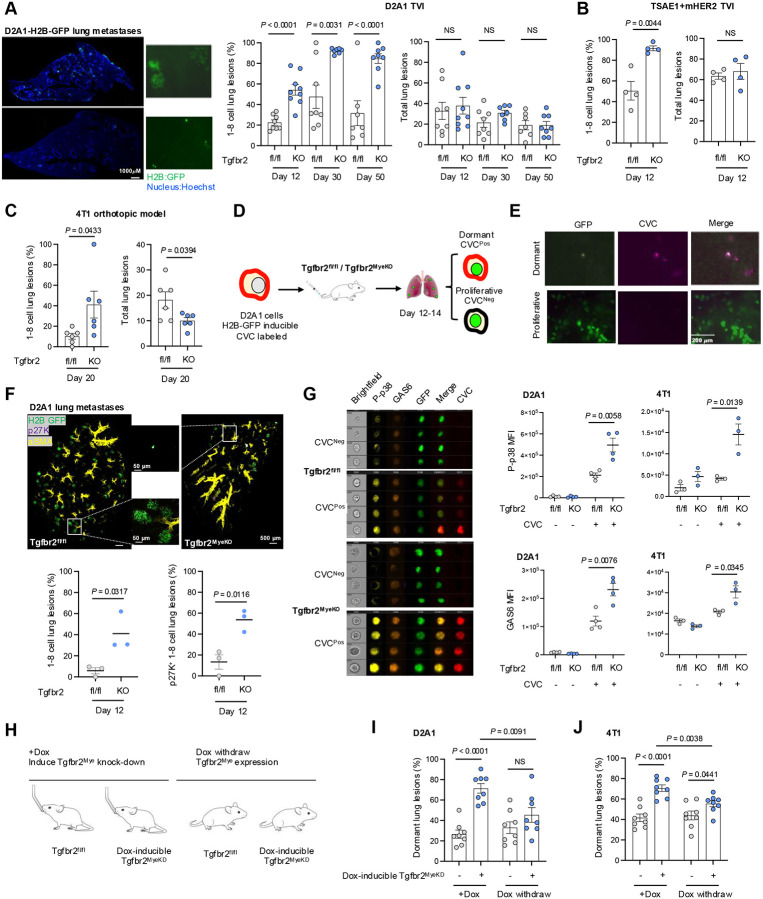
A mouse model of tumor dormancy upon abrogation of myeloid-specific TGF-β signaling **A**. Representative lung surface imaging of metastatic lesions from Tgfbr2^MyeKO^ and Tgfbr2^fl/fl^ (flox control) mice that received D2A1-H2B-GFP TVI for 12–14 days (left), and % dormant lesions (<8 cells) (middle) and total tumor lesions (right) at 12, 30, and 50 days after mice received TVI of tumor cells. n=7–9 mice per group. **B**. Percentage of 1–8 cell tumor lesions (left) and total tumor lesions (right) from the TSAE1+mHer2 TVI model, imaging on day 12 after inoculation. n=4 mice per group. **C**. % 1–8 cell tumor lesions (left) and total tumor lesions (right) from 4T1 orthotopic model, imaging on day 20. n=6 mice per group. **D**. Schematic design for sorting dormant (GFP+CVC^Pos^) and proliferative D2A1-H2B-GFP (GFP+CVC^Neg^) cells. **E**. Representative lung surface images of dormant (GFP+CVC^Pos^) and proliferative lesions (GFP+CVC^Neg^) from mice that received TVI of D2A1-H2B-GFP for 12 days. **F**. Representative Clearing-enhanced 3D (Ce3D) confocal imaging of lung tissues at 12 days after TVI of D2A1-H2B-GFP-mRuby-p27K cells. Green: H2B-GFP, Violet: mRuby-p27, and yellow: aSMA, with enlarged images of boxed regions shown in the middle panels; % 1–8 cell dormant lesions (lower left) and p27K+ dormant lesions (lower right). n=3 mice per group. **G**. Representative imaging flow cytometry of P-p38 and GAS6 in dormant GFP+CVC^Pos^ and proliferative GFP+CVC^Neg^ D2A1 cells from Tgfbr2^MyeKO^ and flox control mice (left) and average of fluorescence intensity of P-p38 and GAS6 in GFP+CVC^Pos^ and GFP+CVC^Neg^ D2A1 and 4T1 cells (right). n=3–4 mice per group. **H**. Schematic design for the effect of Dox-induced myeloid-TβRII-KD and myeloid-TβRII re-expression on dormant lesions. **I-J**. % dormant lesions from TβRII-KD and re-expression after D2A1 TVI (I) and 4T1 orthotopic injection at day 12 (J). n=8 mice per group. All data are presented as mean ± s.e.m. p-values were derived from a two-tailed Student’s t-test and significance was determined by a p-value < 0.05.

We have previously reported that deletion of *Tgfbr2*, the gene encoding TGF-β receptor II (TβRII) in myeloid cells (Tgfbr2^MyeKO^), decreases macroscopic cancer metastasis through increased host anti-tumor immunity^47, 49^. In the current study we employed D2A1, a proliferative isogenic breast cancer cell line that is widely used for tumor dormancy studies^15, 50^ to explore whether such resistance to macroscopic tumor growth involves clearance of cancer cells or induction of dormancy, with the potential for future outgrowth. D2A1 cells were transduced with the Doxycycline (Dox) inducible H2B-GFP system, allowing them to be used in immune-competent mice and for microscopic imaging. Using this model, we find a striking cellular dormancy phenotype upon abrogation of myeloid-specific TGF-β signaling. The dormant tumor lesions are characterized by single cells or cell clusters with fewer than 8 cells (1–8 cell per lesion) in Tgfbr2^MyeKO^ mice 12, 30, and 50 days after tail vein injection (TVI) of D2A1 tumor cells (Figure 1A). This is approximately the equivalent of 1.5–5.5 years in humans, a relevant time window in the context of triple-negative breast cancer relapse (TNBC) and immune checkpoint blockade (ICB) resistance. Consistent with our previous publications, the Tgfbr2^MyeKO^ mice showed fewer gross metastatic nodules and increased survival after TVI with D2A1 cells (Supple fig. S1A-B). The dormancy phenotype was also observed in the TSAE1+mHer2 breast cancer mouse model (Figure 1B) as well as in the 4T1 orthotopic model, an aggressive breast cancer metastatic model where increased numbers of dormant lesions and decreased proliferative lesions were evident in the Tgfbr2^MyeKO^ mice (Figure 1C).

To further characterize dormant lesions from the Tgfbr2^MyeKO^ mice, we labeled D2A1 cells with Cellvue Claret (CVC), a lipophilic dye that is embedded in the plasma membrane and is not degraded during protein turnover but diminishes in intensity upon cell division (Figure 1D). Solitary dormant D2A1 cells or those with less than 2–3 divisions retained CVC labeling, while micro-metastatic lesions did not (Figure 1E). Additionally, we transduced D2A1 cells with the mRuby-p27K quiescence reporter which lacks Cyclin-dependent kinase (CDK) inhibitory activity but retains the susceptibility for ubiquitin-mediated degradation, enabling visualization of cells in the G0 phase. Clearing-enhanced 3D (Ce3D) volume confocal imaging of the lung tissues revealed that approximately 60% of 1–8 cell lung lesions in Tgfbr2^MyeKO^ mice were p27K positive (Figure 1F). Further, imaging flow cytometry analysis revealed higher expression of the previously reported tumor dormancy markers phosphorylated p38 (P-p38) and GAS6, or the two in combination^44, 51, 52^, in CVC^Pos^ dormant cells from Tgfbr2^MyeKO^ mice as compared to those from controls (Figure 1G, and Supple fig. S1D). Based on these data, the 1–8 cell clusters will be referred to as dormant tumor lesions.

We next investigated whether rescue of myeloid TβRII expression would diminish the dormancy phenotype using a Dox inducible myeloid TβRII shRNA knockdown (TβRII-KD) transgenic mouse line (Figure 1H). As expected, Dox-induced myeloid TβRII-KD before D2A1 TVI or 4T1 orthotopic implantation increased the percentage of dormant lesions while withdrawing Dox decreased the frequency of such lesions with no changes in total tumor lesions (Figure 1I, 1J, Supple fig. S1E). In contrast, Dox-induced myeloid TβRII-KD after D2A1 TVI did not increase the frequency of dormant lesions over control (Supple fig. S1F), suggesting the importance of a pre-established distant organ microenvironment in the formation of dormant lesions. There were no changes in the total number of tumors (Supple fig. S1F), indicating that cell death of founder metastatic cells in the lung microenvironment of the Tgfbr2^MyeKO^ mice did not play a major role in these findings. Together, these data further support the value of Tgfbr2^MyeKO^ mice as a relevant *in vivo* model of tumor dormancy using multiple mouse breast cancer cell lines and reveal that while suppressing gross tumor outgrowth, the loss of TGF-γ signaling in myeloid cells promotes survival of metastatic tumor cells in a dormant state.

### SLURP1-mediated induction of the tumor cell quiescent state

**Figure 2.**
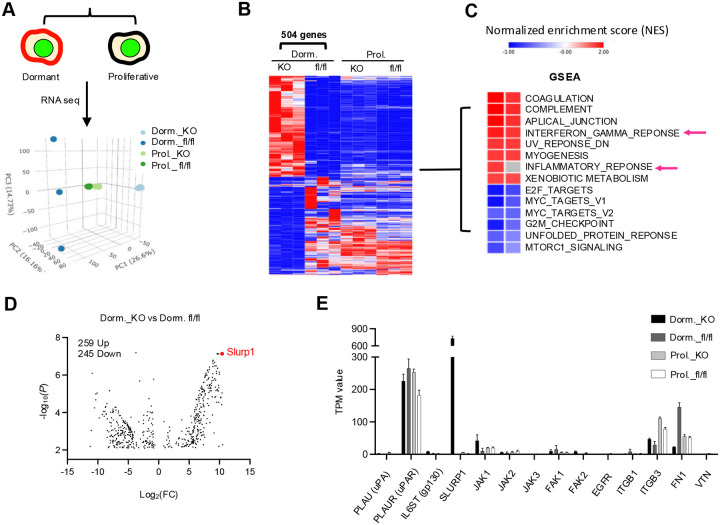
Increased SLURP1 expression in GFP+CVC^Pos^ dormant cells from Tgfbr2^MyeKO^ mice **A**. Schematic for sorting dormant and proliferative tumor cells using inducible H2B-GFP and CVC membrane labeling (left) and PCA plot from RNAseq analysis (right). Dorm: sorted dormant tumor cells; Prol: sorted proliferative tumor cells; flox control. mice; KO: Tgfbr2^MyeKO^ mice. n=3 mice per group. **B**. Heatmap of 504 differential genes from RNAseq data analysis comparing dormant vs proliferative tumor cells from Tgfbr2^MyeKO^ and flox control mice. n=3 mice per group. **C**. Gene set enrichment analysis (GSEA) of differential genes shown in B. **D**. Volcano plot for upregulated and downregulated genes comparing dormant cells from the Tgfbr2^MyeKO^ with those from flox control mice, with SLURP1 (in red) among the top upregulated. **E**. TPM values of genes from RNAseq revealing increased SLURP1 and integrin-mediated pathway including Itgb1, Fn1, uPA and uPAR. n=3 mice per group.

To gain insight into the molecular features of the dormancy state upon abrogation of myeloid TGF-β signaling, we sorted GFP+CVC^Pos^ dormant and GFP+CVC^Neg^ proliferating tumor cells from control and Tgfbr2^MyeKO^ mice 14 days after TVI of D2A1-H2B-GFP cells (Figure 2A, and Supple fig. S2A, S2B, S2C, S2D). RNAseq analysis (biological triplicates: n=50 each for dormant and proliferative cells) showed four distinct cell clusters in the PCA plot (Figure 2A). By comparing dormant and proliferating D2A1 cells from control and Tgfbr2^MyeKO^ mice, we identified 504 differentially expressed genes that were unique to dormant cells from Tgfbr2^MyeKO^ mice (Figure 2B). As expected, gene set enrichment analysis (GSEA) of dormant cancer cells revealed a decreased enrichment of cell cycle mediators including targets of E2F and MYC, as well as mTOR signaling (Figure 2C).

**Figure 3.**
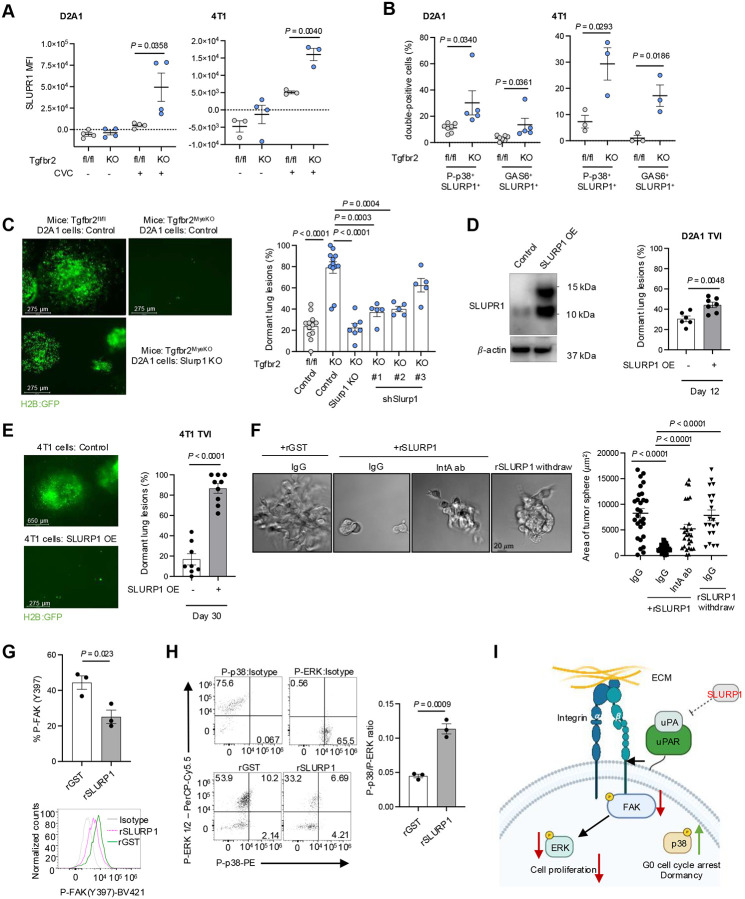
The effect of SLURP1 on tumor dormancy. **A**. SLURP1 fluorescence intensity in dormant GFP+CVC^Pos^ and proliferative GFP+CVC^Neg^ tumor cells from Tgfbr2^MyeKO^ and flox control mice by imaging flow cytometry, D2A1 (left) and 4T1 (right); n=4 biological independent experiments. **B**. % of SLURP1^+^ cells with P-p38^+^ and GAS6^+^ expression from dormant GFP+CVC^Pos^ and proliferative GFP+CVC^Neg^ tumor cells from Tgfbr2^MyeKO^ and flox control mice; n=4 four biological independent experiments. **C**. Representative lung surface imaging (left), and % dormant lesions from D2A1 tumor cells with Slurp1-KO and -KD (right). n=5–12 mice per group. **D**. Western blot for SLURP1-OE in D2A1 tumor cells (D2A1-SLURP1-OE) (left), and % dormant lesions from mouse lungs at 12 days after TVI of D2A1-SLURP1-OE tumor cells (right). **E**. % dormant lesions from mouse lungs at day 30 after TVI of 4T1-SLURP1-OE tumor cells. n=9–10 mice per group. **F**. Representative images of D2A1 spheroid culture treated with recombinant SLURP1 (rSLURP1), and rSLURP1 plus an integrin activating antibody (IntA ab), as well as with rSLURP1 withdraw (left) and quantification of spheroid size (right). **G-H**. Flow cytometry analysis of P-FAK (G), P-p38, P-ERK, and the ratio of P-p38 to P-ERK (H) in 3D-cultured D2A1 cells treated with rSLURP1 or GST control. **H**. Schematic for SLURP1 mediated inhibition of tumor cell proliferation (created with BioRender.com). All data are presented as mean ± s.e.m. p-values were derived from a two-tailed Student’s t-test. Significance was determined by a p-value< 0.05.

Slurp1 was one of the most abundant transcripts in dormant cells from Tgfbr2^MyeKO^ mice (Figure 2D and 2E). SLURP1, a secreted Ly6/uPAR related protein, disrupts uPA (urokinase plasminogen activator)-mediated cell proliferation in corneal homeostasis^53, 54^ and shows anti-proliferation properties in cancer^55^. Imaging flow cytometry revealed a higher expression of SLURP1 in CVC^Pos^ D2A1 and 4T1 dormant cells from Tgfbr2^MyeKO^ mice as compared to tumor cells from control mice (Figure 3A). These SLURP1^+^CVC^Pos^ cells also showed high levels of P-p38 and GAS6 (Figure 3B and Supple fig. S3A), consistent with our findings above.

To investigate the contribution of SLURP1 to the dormant state we generated Slurp1 knock-out (Slurp1-KO) and Slurp1 shRNA knockdown (Slurp1-KD) D2A1 cells (Supple fig. S3B, S3C). Tgfbr2^MyeKO^ mice injected with Slurp1-KO or -KD tumor cells showed fewer dormant lesions (Figure 3C) with only a modest effect on the total tumor lesion number (Supple fig. S3D). Conversely, SLURP1 overexpression (SLURP1-OE) in D2A1 and 4T1 cell lines increased the percentage of dormant lesions in wild-type mice (Figure 3D, 3E and Supple fig. 3E), accompanied by decreased total lesions (D2A1) or no difference in lesion number (4T1) (Supple fig. S3F). Consistently, D2A1 with SLURP1-OE decreased the number of gross metastatic nodules quantified by Indian Ink staining (Supple fig. S3G). In an *in vitro* 3D culture model, recombinant SLURP1 treatment decreased D2A1 spheroids size in a dose-dependent manner (Supple fig. S3H).

SLURP1 has been reported to interact with uPA^53^ and uPA-uPAR interaction is critical to the activation of integrin-FAK-ERK signaling pathways and promotion of tumor cell proliferation^52, 56, 57^. When an integrin-activating antibody (IntA ab) was added to the D2A1 spheroid culture, it reversed the inhibitory effect of recombinant SLURP1 on spheroid growth (Figure 3F), and as expected, withdrawing SLURP1 treatment rescued spheroid growth (Figure 3F). This connection of SLURP1 expression to adhesion and tumor growth was also supported by RNAseq analysis of tumor spheroids treated with recombinant SLURP1 compared with GST control, which showed integrin cell surface interactions and extracellular matrix organization as the top enriched pathways (Supple fig. S3I). Further, recombinant SLURP1 treatment decreased FAK phosphorylation (Figure 3G) and increased the P-p38 to P-ERK ratio (Figure 3H), characteristic of dormant cancer cells^58^. Together with previous findings in the literature, our *in vivo* and *in vitro* data support an inhibitory role of SLURP1 in tumor cell proliferation by targeting the integrin-FAK-ERK signaling pathways (Figure 3I).

### IFNγ-KLF4 axis regulation of SLURP1 expression

**Figure 4.**
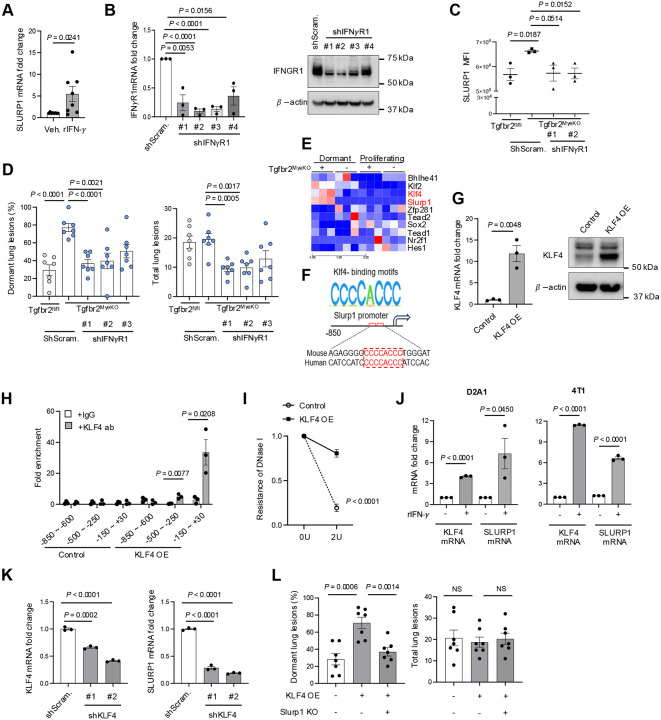
IFN-γ-KLF4 axis regulation of SLURP1 expression. **A**. SLURP1 expression in cultured tumor cells treated with IFN-γ. n=8, independent biological replicates. **B**. RT-qPCR and Western blot validation of IFN-γR-KD in D2A1 cells. **C**. SLURP1 expression from Imaging flow cytometry of dormant cells with or without IFN-γR-KD, single cell suspension from the lungs of the tumor-bearing mice. n=3 biologically independent experiments. **D**. Dormant (left) and total tumor lesions (right) from lung surface imaging of mice that received D2A1 cells with or without IFN-γR-KD. n=7–8 mice per group. **E**. TPM expression heatmap of KLF4 transcription family members from RNAseq of the dormant cells. n=3 mice per group. **F**. KLF4 binding site mapping in the *Slurp1* promoter. Putative KLF4-binding motifs were predicted in the mouse *Slurp1* promoter(up) by Homer assay. Klf4 binding motif CCCCACCC were shown in mouse and human *Slurp1* promoter. **G**. RT-qPCR and Western blot of KLF4-OE in D2A1 cells. **H**. Fold enrichment of SLURP1 expression from KLF4-ChIP qPCR of D2A1 cells, n=3 biologically independent experiments. Results shown as mean ± SD. **I**. DNase-mediated DNA degradation showing KLF4-OE protected the KLF4 binding regions in *Slurp1* promoter. n=3 biologically independent experiments. Results are shown as mean ± SD. **J**. RT-qPCR of KLF4 and SLURP1 expression in cultured tumor cells treated with recombinant IFN-γ (left: 4T1, and right: D2A1). **K**. The effect of KLF4-KD (left) on fold changes of SLURP1 expression (right) in D2A1 cells. n = 3 experiments. **L**. Dormant (left) and total tumor lesions (right) from lung surface imaging of mice that received TVI of D2A1 tumor cells with KLF4-OE, as well as D2A1 cells with KLF4-OE plus Slurp1-KO. n=7 for each group. Data are presented as mean ± s.e.m. p-values were derived from a two-tailed Student’s t-test. Significance was determined by a p-value< 0.05.

What features of the tumor microenvironment in Tgfbr2^MyeKO^ mice promoted the increased expression of SLURP1 that limits tumor proliferation? Examination of differentially expressed genes comparing Tgfbr2^MyeKO^ dormant tumor cells vs. those from control mice suggested that dormancy may arise from signals within an activated immune microenvironment dependent on myeloid-specific *Tgfbr2* deletion (Figure 2C, red arrows). Further, a Bio-Plex immunoassay detected higher levels of IFN-γ as well as TNF-α in the lung microenvironment of tumor-bearing Tgfbr2^MyeKO^ mice as compared to control mice (Supple fig. S4A). Recombinant IFN-γ treatment of D2A1 tumor cells increased SLURP1 mRNA *in vitro* under stress and nutrient-deprived conditions (Figure 4A). Importantly, KD of the IFN-γ receptor (IFN-γR) decreased SLURP1 expression in D2A1 cells (Figure 4B and C), consistent with a decreased pSTAT1 down stream of IFN-γ signaling (Supple Fig. S4B). This was associated with decreased P-p38 and GAS6 protein expression in CVC^Pos^ D2A1 cells from Tgfbr2^MyeKO^ mice (Supple Fig. 4C). IFN-γR-KD in D2A1 cells also decreased the percentage of dormant lesions in Tgfbr2^MyeKO^ mice (Figure 4D). These data suggest the importance of IFN-γ signaling in promoting SLURP1 expression and tumor dormancy induction.

Using data from our RNAseq analysis comparing dormant and proliferating cells, we next looked for differential expression of transcription factors that might regulate SLURP1 expression and found KLF4 and KLF2 but not other Krüppel-like factor family members were increased in D2A1 cells from Tgfbr2^MyeKO^ mice (Figure 4E). The Homer assay predicted a putative KLF4-binding motif CCCCACCC to be present in mouse and human Slurp1 promoters (Figure 4F). KLF4-ChiP assays in D2A1 tumor cells with KLF4-OE (Figure 4G) revealed that KLF4 protein was enriched at the −500~−250 and −150~+30 bp regions of the SLURP1 promoter (Figure 4H), consistent with this motif mapping. Moreover, the KLF4 DNA binding sites in the SLURP1 promoter were protected from DNA degradation in D2A1 dormant cells (Figure 4I). In accord with these findings, both KLF4 and SLURP1 were increased in 4T1 and D2A1 tumor cells upon treatment with IFN-γ (Figure 4J). Additionally, shRNA KLF4-KD decreased SLURP1 expression and KLF4-OE increased it in D2A1 cells (Figure 4K and Supple fig. S4D). In wild-type mice, the D2A1 cells with KLF4-OE showed increased numbers of dormant lesions and Slurp1-KO in these KLF4-OE cells reversed the dormancy phenotype (Figure 4L), indicating SLURP1 indeed serves as a downstream mediator of KLF4’s dormancy effect. Together, these results suggest a critical role of an IFN-γ-KLF4-SLURP1 axis in the observed tumor dormancy phenotype.

### Elevated IFN-γ levels in metastatic lung tissue and immune niches of the Tgfbr2^MyeKO^ mice,

**Figure 5.**
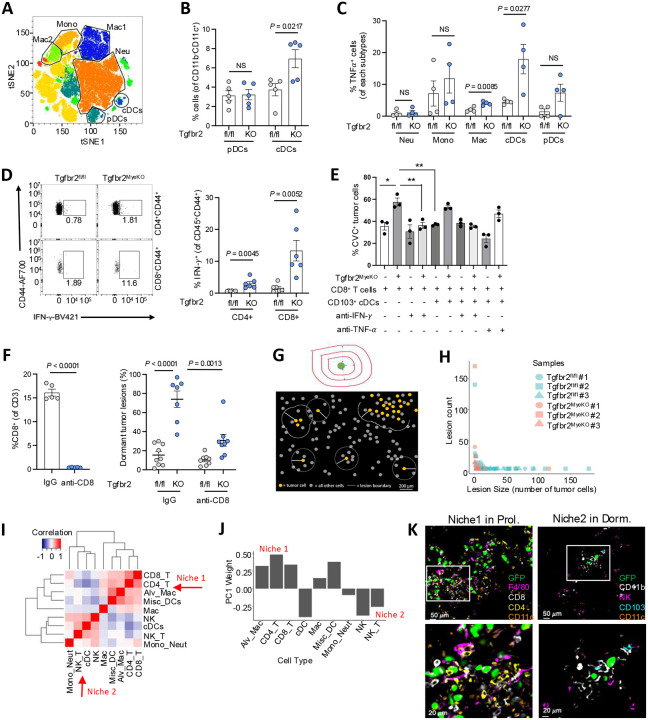
Immune cell profiling and immune niche characterization of the lungs from tumor-bearing Tgfbr2^MyeKO^ and control mice. **A**. t-SNE plots for all myeloid cells by Cytek analysis. **B**. % CD103^+^ DCs and plasmacytoid DCs (pDCs) in CD45^+^CD11b^−^ population. **C**. %TNF-α^+^ neutrophils, monocytes, macrophages, CD103^+^ DCs and pDCs. n=5–6 mice per group. **D**. Flow cytometry analysis of IFN-γ^+^CD4^+^ and IFN-γ^+^CD8^+^ T cells (gated on CD44^+^ effector cells) from the lung of Tgfbr2^MyeKO^ and flox control mice. n=7 mice per group. **E**. Percent of dormant D2A1 cells (CVC^Pos^) in a co-culture of tumor cells with CD8^+^ T and CD103^+^ DCs with IFN-γ or TNF-α neutralizing antibodies (n=3 biological replicates). **F**. Flow cytometry validation of CD8^+^ T cell depletion from the lung of Tgfbr2^MyeKO^ mice (left). Dormant tumor lesions from the lungs of Tgfbr2^MyeKO^ and flox control mice upon CD8^+^ T cell depletion (right). n =7 mice per group. **G**. Strategy for demarcating individual tumor lesions separated by 200 μm distance to define neighbors. n=3 each for Tgfbr2^MyeKO^ and flox control mice. **H**. Distribution of tumor lesion sizes from each mouse lung sample. Lesions in flox control mice are larger on average (zero-truncated negative binomial regression model, *P* = 0.0028). **I**. Pairwise correlations of standardized center log ratios (CLRs) for each immune cell type across all tumor lesions. Two immune niches of commonly co-occurring cell types are outlined and indicated. **J**. Loadings for PC1 of the PCA, where a positive weight indicates increasing fractional abundance of a cell type as PC1 increases, and a negative weight indicates decreasing fractional abundance of a cell type as PC1 increases. **K**. Representing images showing niche 2 (CD103^+^DCs, NK cells Monocyte/Neutrophils) surrounding dormant tumor lesions in Tgfbr2^MyeKO^ mouse lungs and niche 1 (alveolar macrophages, CD8^+^ and CD4^+^ T cells) surrounding proliferative tumor lesions. All data are presented as mean ± s.e.m. p-values were derived from a two-tailed Student’s t-test. Significance was determined by a p-value< 0.05.

Myeloid *Tgfbr2* deletion alters immune cell function, with a significant impact in both tumor-bearing mice and stroke occurrence in aging mice^47, 59^. To better understand the way in which the *Tgfbr2* deletion affected local immune events that contributed to the observed tumor dormancy phenotype, we used spectral flow cytometric profiling of immune cells in the lungs of tumor-bearing Tgfbr2^MyeKO^ and control mice. The primary change among myeloid cells was an increase in CD103^+^ dendritic cells (DCs) (Figure 5A and 5B), with no difference in the overall number of CD45^+^ immune cells (Supple fig. S5A, S5B). In addition, the expression of TNF-α was significantly higher in CD103^+^ DCs from tumor-bearing Tgfbr2^MyeKO^ mice (Figure 5C). In the lymphoid compartment, tumor-bearing Tgfbr2^MyeKO^ mice showed a substantial increase in IFN-γ^+^CD4^+^ and especially IFN-γ^+^CD8^+^ T cells, and a more modest but significant increase in IFN-γ^+^ NK cells (Figure 5D, Supple fig. S5C). These data suggest an increase in type 1 (inflammatory) immunity at the metastatic lung in the Tgfbr2^MyeKO^ mice. To further assess the role(s) of IFN-γ and TNF in the induction of dormancy, a coculture of CVC labeled D2A1 tumor cells with T and DCs from Tgfbr2^MyeKO^ mice was carried out. Interestingly, neutralizing IFN-γ but not TNF-α, decreased CVC^Pos^ and increased Ki-67^+^ D2A1 cells (Figure 5E, Supple fig. S5D). To directly interrogate the roles of CD8^+^ T in driving the dormancy phenotype in Tgfbr2^MyeKO^ mice, we performed CD8^+^ T cell depletion. As expected and consistent with our previous findings^47^, CD8^+^ T cell depletion increased proliferative lesions and concurrently diminished the Tgfbr2^MyeKO^ tumor dormancy phenotype (Figure 5F, and Supple fig. S5E). In contrast, NK depletion did not show a major effect on dormancy phenotype like that seen with CD8^+^ T depletion (Supple fig. S5F). Together, these results suggest IFN-γ production from CD8^+^ T cells is likely to be the primary drivers of the observed dormant tumor phenotype.

cDC1 and CD8^+^ T cells are primary components in the TME of effective adaptive anti-tumor responses, especially upon ICB immunotherapy; their possible involvement in promoting development of immune-resistant dormant tumor cells responsible for late arising metastatic lesions in patients with apparent remissions is thus a critical issue to examine, as it could reveal a duality to the effects of immunotherapy. We therefore next asked whether dormant tumor lesions resided in a distinct cellular niche where they escaped immune clearance as compared to sites of tumor cell proliferation. We employed IBEX multiplex immunostaining/imaging technology^60^ (Table 3) and a custom pipeline for defining tumor lesions and their immune niches (Figure 5G). We segmented and classified all cells in the IBEX images based on known markers, defined lesions as groups of tumor cells separated by no more than 200μm and included all immune cells within 200μm of any tumor cell as part of that lesion (Figure 5G and Supple fig. S6A-B). We identified 183 tumor lesions, 115 from control and 68 from Tgfbr2^MyeKO^ mice. As expected and consistent with the dormancy phenotype upon abrogation of myeloid TGF-β signaling, tumor lesions were significantly larger in control mice (*P* = 1.5*10^−10^), containing an average of 16.8 tumor cells compared to an average of 3.8 tumor cells in the lesions of Tgfbr2^MyeKO^ mice (Figure 5H, Supp Figure S6C). Next, we used pairwise abundance correlations and principal component analysis (PCA) (Figure 5I and J) to study the immune cell compositions of the tumor lesions. These approaches independently uncovered two distinct immune niches: Niche 1 contains CD4^+^ and CD8^+^ T cells, alveolar macrophages, macrophages, and diverse DCs; surprisingly, these sites containing the T cells shown above to be the major producers of IFN-γ were associated with small proliferative lesions rather than cellular dormancy; conversely, Niche 2 was enriched in CD103^+^ cDCs, NK cells, NKT cells, and monocytes/neutrophils (Supp Figure S6D and S6E) and associated with true tumor cell dormancy. Smaller tumor lesions tended to have Niche 2-like immune cell compositions (*P* = 0.0006), as did lesions from Tgfbr2^MyeKO^ mice (*P* = 0.0190), while larger tumor lesions and tumor lesions from control mice tended to have Niche 1-like immune cell compositions (Supp Figures S6D, S6E and S6F, shown visually in Figure 5K). These spatial findings suggest a series of events are involved in the generation of the IFN-γ that induces dormancy, which in turn protects tumor cells from immune attack, and constrains tumor lesions from proliferative expansion. We propose a model in which a subset of metastatic tumor cells able to be rapidly induced to dormancy by IFN-γ upon seeding to the lung (possibly produced at a distance by activated CD8^+^ T cells) escape destruction by nearby NK cells, whereas those tumor cells that avoid induction of dormancy by IFN-γ proliferate but are kept as small proliferating lesions by persistent cytotoxic action of the colocalized CD8^+^ T cells.

### CD200-CD200R1 mediated immune evasion of dormant tumor cells

**Figure 6.**
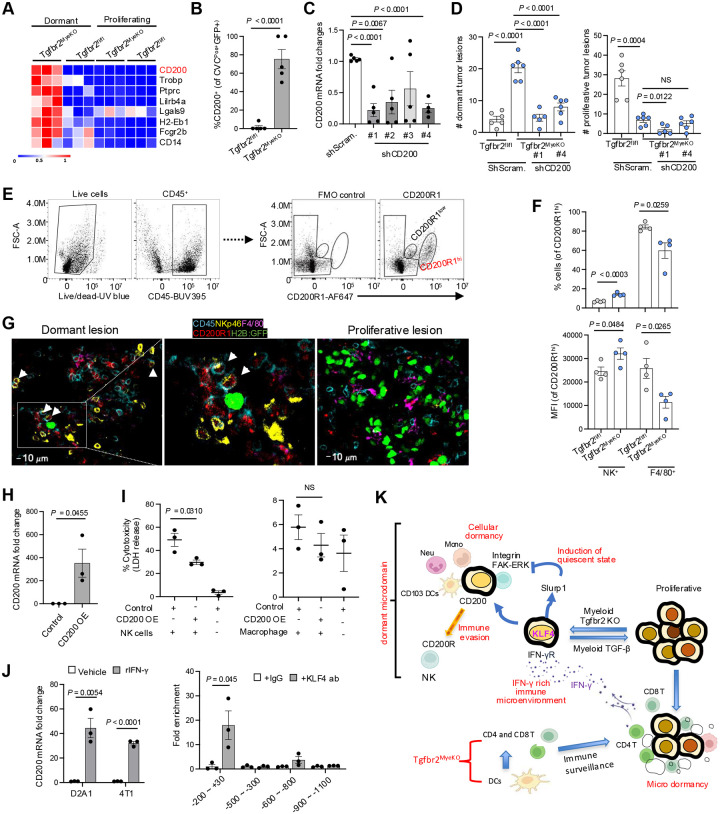
Mechanisms of immune evasion by dormant tumor cells **A**. Heatmap of the immune evasion genes enriched from the dormant tumor cells from the Tgfbr2^MyeKO^ mice. n=3 mice per group. **B**. % CD200^+^ cells in CVC^Pos^ dormant tumor cells by Cytek analysis. n=5 mice per group. **C**. RT-qPCR for validation of CD200-KD in D2A1 cells. n=4 biological replicates. **D**. Number of dormant lesions (left) and proliferative tumor lesions (right) from D2A1 cells with CD200-KD and control. n=5–6 mice per group. **E**. Flow cytometry gating of CD200R1^+^ immune cells (left), and CD200R1^Hi^ and CD200R1^Low^ subclusters (right panels). **F**. Percentage (left) and mean immunofluorescence intensity (right) of CD200R1^Hi^ in NK and macrophage cells comparing Tgfbr2^MyeKO^ and flox control mice. n=4 mice per group. **G**. Representative images of CD200R1^+^ NK and macrophages in the proximity of dormant and proliferative metastatic lesions. **H**. RT-qPCR validation of CD200-OE in D2A1 tumor cells. **I**. % cytotoxic of CD200R1^+^ NK (left) and macrophage cells (right) co-cultured with D2A1 tumor cells with CD200-OE. n=3 biological replicates. **J**. left: RT-qPCR CD200 mRNA fold change in cultured D2A1 and 4T1 tumor cells in response to IFN-γ treatment; right: fold enrichment of CD200 expression from KLF4-ChIP qPCR of D2A1 cells with KLF4-OE. n = 3 biologically independent experiments. Results are shown as mean ± SD. All data are presented as mean ± s.e.m. p-values were derived from a two-tailed Student’s t-test. Significance was determined by a p-value< 0.05. **K**. Schematic hypothesis for immune-mediated dormancy upon abrogation of myeloid TGF-β signaling: myeloid *Tgfbr2* deletion enhances cDC function and activates CD4^+^ and CD8^+^ T cells leading to an IFN-γ rich tumor microenvironment. IFN-γ-induces KLF4-mediated SLURP1 expression which blocks the Integrin-FAK-ERK and promotes tumor cell quiescent state. Within the dormant microdomain, the quiescent tumor cells upregulate the immune evasion program through KLF4-CD200-CD200R mediated inactivation of NK cells.

To better dissect this complex interplay between immune cells and tumor state, we focused on possible evasion of killing of dormant cells in Niche 2 containing many NK cells. Our RNAseq data revealed several potential immune mediators and regulatory pathway candidates (Figure 6A, Supple fig. S7A and S7B). Of these, high levels of CD200 mRNA were particularly evident in dormant tumor cells from Tgfbr2^MyeKO^ mice (Figure 6A) and the elevated expression was further validated at the protein level by spectral flow studies (Figure 6B, and Supple fig. S7C). CD200 is a membrane glycoprotein structurally related to B7 family that signals through CD200R1, promoting immune tolerance and NK inactivation^61–63^. To see if this inhibitory pathway contributed to immune evasion by the dormant tumor cells, we used shRNA to knock-down expression of CD200 in D2A1 tumor cells (Figure 6C) and observed fewer dormant lesions when these cells were injected into Tgfbr2^MyeKO^ mice (Figure 6D), consistent with a role for CD200 in dormant tumor cell resistance to immune effector function.

To further dissect mechanism(s) of immune evasion mediated by CD200, we focused on CD200R1^Hi^ immune cells which included the NK cells and macrophages. Only NK cells but not macrophages displayed an increased cell number and CD200R1^Hi^ phenotype when comparing cells recovered from Tgfbr2^MyeKO^ with those from control mice (Figure 6E and F). A very small number of CD3 T cells were CD200R1^Hi^ but no difference was found in the percentage or MFI of these T cells between Tgfbr2^MyeKO^ and WT mice (Supple fig. S7D). To probe the potential function of CD200 in regulating NK activity, we set up an *in vitro* co-culture of CD200-overexpressing D2A1 tumor cells (Figure 6H) with CD200R1^Hi^ NK or CD200R1^Hi^ macrophages as a control. There was a clear decrease in the cytotoxicity induced by CD200R1^Hi^ NK cells (Figure 6I, left panel) but not by macrophages (Figure 6I, right panel). These data indicate one mechanism by which dormant tumor cells escape immune clearance is inhibition of NK cell-mediated cytotoxicity through CD200-CD200R1 interactions.

Connecting these data with our earlier observations on the role of IFN-γ in promoting dormancy, we found increased CD200 expression in cultured D2A1 and 4T1 tumor cells in response to IFN-γ treatment (Figure 6J, left panel). Since IFN-γ induced KLF4 expression, we looked for KLF4 binding sites in both mouse and human CD200 promoters and indeed saw the presence of the KLF4 binding motif CCCCACCC in these promoters using the Homer assay (Supple fig. S7E). Further, KLF4-ChiP assays of the CD200 promoter in D2A1 tumor cells with KLF4-OE revealed that KLF4 protein was enriched at −200~+30bp region of the CD200 promoter (Figure 6J, right panel). Together, the findings suggest that myeloid *Tgfbr2* deletion enhances CD103^+^ DC function that activates CD4^+^ and CD8^+^ T cells leading to an IFN-γ rich tumor microenvironment. IFN-γ in turn induces KLF4-mediated expression of SLURP1 that is critical in promoting tumor dormant state. These data fit with the previous conclusion that dormancy is induced by IFN-γ and these cells escape NK-mediated killing, which we now can ascribe in large measure to KLF4-mediated upregulation of an immune evasion program involving CD200-CD200R1 (Figure 6K).

### Human correlative studies of tumor dormancy and treatment relapse, targeting the CD200-mediated dormant niche

**Figure 7.**
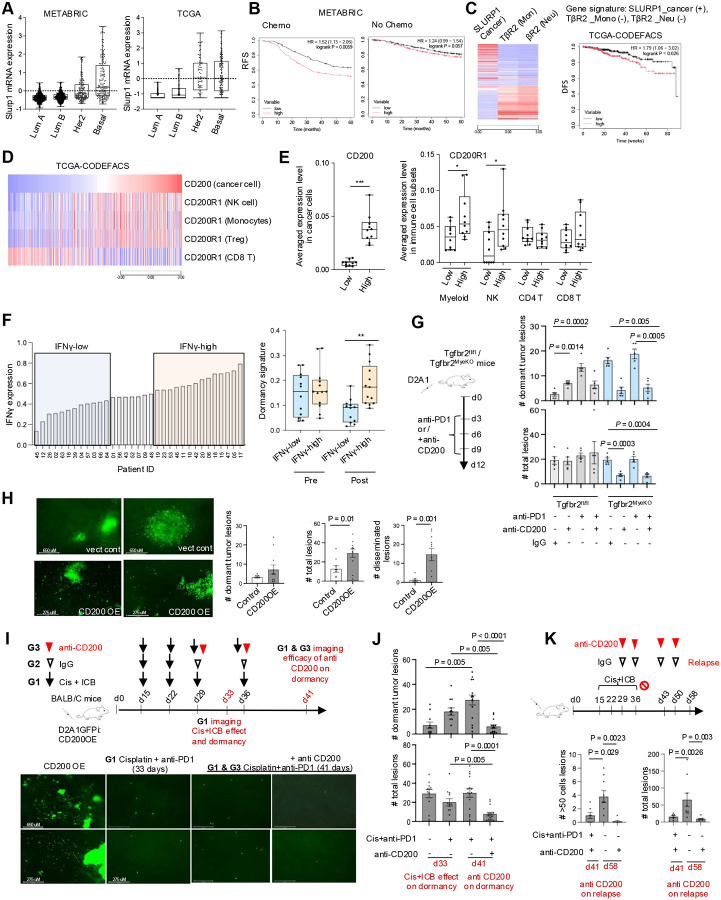
Human correlative studies of SLURP1, TβRII, and CD200-CD200R, and targeting CD200 mediated dormant niche. **A**. SLURP1 expression in four subtypes of breast cancer from METABRIC and TCGA datasets. **B**. Kaplan-Meier relapse-free survival (RFS) of SLURP1 mRNA expression in breast cancer patients with (left) or without chemotherapy (right). **C**. Heatmap of the SLURP1expression in cancer cells and TβRII expression in monocytes and neutrophils; and their correlation with Kaplan-Meier disease-free survival (DFS), TCGA CODEFACS, deconvoluted datasets with cell type identification (https://zenodo.org/record/5790343). **D**. Heatmap analysis of CD200 in cancer cells, CD200R1 in CD56 NK, CD14 monocytes, Treg cells, and CD8^+^ T cells from the TCGA-CODEFACS human breast cancer dataset, with low in blue, high in red. **E**. Correlation of CD200 in cancer cells with CD200R1 in myeloid, NK, CD4^+^ and CD8^+^ T cells. Patients were grouped by average CD200 expression in cancer cells. **F**. Correlation of IFN-γ levels (patients divided by NK/T cell IFN-γ expression into high and low IFN-γ groups, left panel) with the dormancy signature in breast cancer before and after radiotherapy and anti-PD1 treatment (Cancer Cell 2024 dataset). **G**. Proof of principle experimental design for the effect of targeting CD200 on tumor dormancy (left), number of dormant (top right) and total tumor lesions (lower right) from Tgfbr2^MyeKO^ and flox control mice treated with anti-CD200 alone or in combination with anti-PD1. n=5 mice per group. **H**. The effect of CD200-OE on dormant tumor cells (left), metastatic nodules (middle) and disseminated tumor cells (right). **I-J**. Schematic design of targeting CD200 in combination with Cisplatin+anti-PD1 in BALB/C mice injected with CD200OE tumor cells and representative IF images (I); number of dormant and total lesions treated with Cisplatin+anti-PD1, or Cisplatin+anti-PD1+ anti-CD200 on day 33 and 41 (J). n=12–16 mice per group. **K**. The effect of anti-CD200 in mouse models of ICB relapse (schematic), the mice were treated with standard chemo + ICB until day 36, followed by treatment break until day 58. Bar figure showing the number of macro- or total tumor lesions in the lungs of mice treated with anti-CD200 or IgG2a as indicated. n=7–9 mice per group. p-values were derived from a two-tailed Student’s t-test for box. Statistical significance was determined by log-rank test for survival analysis.

We next investigated the clinical relevance of SLURP1 and CD200 in breast cancer patient datasets. SLURP1 expression was the highest in Basal and Her2+ forms of the four breast cancer subtypes in both METABRIC and TCGA datasets (Figure 7A). High SLURP1 but not GAS6, a published dormancy marker, predicted a decreased relapse-free survival (RFS) for patients treated with chemotherapy but not those without treatment (Figure 7B, Supple fig. 8A). We further examined the correlation of patient survival with an increased SLURP1 in cancer cells and a decreased TβRII in monocytes and neutrophils (Figure 7C, left). Computationally deconvolving the TCGA-BRCA dataset^64^ revealed that a signature of high Tu-SLURP1 and negatively weighted Mye-TβRII correlated with decreased survival (Figure 7C, right). There was no such correlation when survival was examined with respect to SLURP1 in cancer cells alone, or TβRII in monocytes alone, or TβRII in neutrophils alone (Supple fig. 8B). These data suggest a potential role of SLURP1 and myeloid TβRII in tumor dormancy and its association with treatment relapse.

The critical role of the CD200-CD200R1 in immune evasion of dormant tumor cells led us to examine its relevance in breast cancer patients. Using the deconvolved TCGA-BRCA dataset, we found that patients with high CD200 levels in cancer cells showed positive correlation with CD200R1 in NK cells, CD14+ myeloid cells, and Treg but not CD8^+^ T cells, suggesting that CD200-CD200R1-mediated immune evasion is unlikely a mechanism that affects CD8^+^ T cell anti-tumor responses. (Figure 7D). These results were further supported by another analysis of single cell RNAseq (scRNAseq) dataset of a human breast cancer^65^(Figure 7E). We next generated a dormancy gene signature that included SLURP1, IFNGR1, IFNGR2, KLF4, and CD200 in cancer cells along with CD200R1 in NK and T cells. We evaluated its association with immunotherapy responses using publicly available human breast cancer scRNAseq datasets. We divided the patients into IFN-γ high and IFN-γ low groups based on IFN-γ expression in NK and T cells (Figure 7F, left panel). High IFN-γ levels were correlated with high expression of the dormancy signature after anti-PD1 treatment, but not before the treatment^66^ (Figure 7F). Similar result was observed in another scRNAseq dataset with combination treatment of radiotherapy and anti-PD1^65^ (Supple fig. 8C). Together, these human correlative studies reveal a tumor dormancy phenotype in response to increased IFN-γ anti-tumor immune response, supporting a duality of ICB that we observed in Tgfbr2^MyeKO^ mouse models.

We next targeted the CD200 mediated dormant niche using a CD200 neutralizing antibody. For proof of principle, we first tested the anti-CD200 effect on tumor dormancy (Figure 7G). Both anti-CD200 alone or in combination with anti-PD1 eliminated dormant cancer cells in Tgfbr2^MyeKO^ mice (Figure 7G, Supple fig. S8D), suggesting that the CD200-mediated dormant niche could be therapeutically targeted. To recapture the dormant niche observed in the Tgfbr2^MyeKO^ mice, we developed D2A1 cells with CD200-OE and used them in a preclinical model in immune competent mice. Not surprisingly, the D2A1-CD200-OE cells gave rise to an increased number of dormant tumor cells, significantly more disseminated tumor cells and metastatic nodules than the vector control (Figure 7H). We then investigated the efficacy of anti-CD200 on dormant tumor lesions (Figure 7I, schematic). The combination of cisplatin and anti-PD1 (simulating the clinical standard of care) eliminated most proliferative tumor cells and nodules but left behind dormant cells when assessed on day 33 (Figure 7I, schematic, IF panels, and Figure 7J). Such cells were markedly diminished in number upon addition of anti-CD200 when assessed on day 41 (Figure 7I, schematic and IF panels, Figure 7J, Supple fig. S8E). Not surprisingly, anti-PD1 + anti-CD200 treatment increased Granzyme B and Perforin double positive NK cells (Supple fig. S8F). Importantly, in mice that developed metastatic relapse after 3-week break of chemo + ICB treatment (Figure 7K schematic), the anti-CD200 ab alleviated the metastatic relapse (Figure 7K). Our studies show that anti-CD200 could be utilized to target dormant niche, metastasis relapse and immune evasion, one of the most predominant contributors to patient fatalities and a key challenge in cancer treatment and patient fatality.

## Discussion

Our findings demonstrate that dormant tumor cells persist over long periods by co-opting conserved growth arrest and immune evasion mechanisms regulated by the innate and adaptive limbs of the immune system. Abrogation of myeloid-specific TGF-β signaling induced tumor dormancy by promoting an IFN-γ rich tissue microenvironment that activates KLF4-mediated cellular reprogramming. The IFN-γ-KLF4-SLURP1 axis promotes a quiescent state through inhibition of integrin-FAK-ERK signaling, as well as a KLF4-CD200 axis that leads to immune evasion through engagement of CD200R1^+^ on NK cells.

These insights may provide an explanation for post-ICB relapse in which the effective adaptive anti-tumor responses are dependent on cDC1 and CD8^+^ T cells, with this same response leading to the development of immune-resistant dormant tumor cells due to an increase in local IFN-γ. These dormant tumor cells are presumably responsible for late-arising metastatic lesions in patients with apparent remissions. Of great relevance and importance, our studies reveal that anti-PD1 ICB mostly eliminate proliferative metastatic cancer cells but leaving dormant tumor cells untouched. These cells were effectively targeted by a combination of ICB and anti-CD200 therapy, suggesting a strategy for preventing dormancy-mediated relapse. The immune evasion mechanism involving CD200-CD200R1 mediated inactivation of NK cells in the dormant niche adds to the existing body of work showing cellular dormancy is related to evasion of CD8^+^ T cell– and NK cell-mediated detection and clearance of the tumor cells^21, 36, 37, 39,62, 67^.

The tumor dormancy phenotype in our Tgfbr2^MyeKO^ mice is striking in showing that a single gene deletion in immune cells can induce tumor dormancy *in vivo*. Given the scarcity of suitable mouse models, our system advances the study of immune-regulated tumor dormancy beyond traditional paired proliferative and non-proliferative tumor cell line comparisons such as 4T1/4T07 and D2A1/D2.OR^5, 44, 45^. It is worth noting the observed shift in dormant versus proliferative lesions in our mutant animals despite stable total lesions counts, revealing the pivotal role of host immunity in tumor dormancy regulation.

Many papers in the literature associate various mediators in the TGF-β pathway with regulation of tumor dormancy, including BMP, TGF-β2, and TGF-βRIII^23, 43, 68^. Our previous studies and the present report specifically point to the critical role of myeloid cell TGF-β signaling in immune regulation of tumor dormancy. Tumor-associated myeloid cells are abundant in the tumor stroma and immune organs. These cells include neutrophils, tumor-associated macrophages (TAMs), dendritic cells (DCs), and monocytic and granulocytic myeloid-derived suppressor cells (MDSCs). The latter in particular contribute to compromised host immune surveillance and cancer recurrence^69, 70,8, 71–78^. Our previous studies demonstrated that myeloid TGF-β signaling is critical in immune/inflammatory homeostasis as its abrogation inhibited tumor metastatic progression and its prolonged absence also induced spontaneous stroke in aged mice^47–49, 59^. Together with the results presented here, we propose that myeloid TGF-β signaling acts as a switch: the “ON” mode promotes immune suppression and metastatic outgrowth, while the “OFF” mode fosters anti-tumor immunity and tumor dormancy.

Furthermore, our study revealed two distinct spatially localized immune niches: Niche 1 populated with CD4^+^ and CD8^+^ T cells, alveolar macrophages, macrophages, and various DCs associated with proliferative tumor lesions and Niche 2 enriched with CD103^+^ cDCs, monocytes, neutrophils, NK cells surrounding tumor cells with the characteristics of cellular dormancy. These findings reveal critical cellular and molecular mechanisms for the induction of tumor cell quiescence and immune evasion, two of the most critical tumor dormancy hallmarks, while highlighting the complex spatiotemporal changes that lead to a mix of cellular dormancy and tumor growth control. Indeed, multiple immune cells play roles in dormant niche formation and tumor cell emergence from dormancy, including neutrophils^21, 79, 80^, macrophages^77, 78^, DCs^12^, CD4^+^ T^81^ and CD8^+^ T cells, and NK cells^12, 29, 36, 38, 39, 82, 83^. However, the mechanism underlying cellular dormancy regulated by host immunity *in vivo* is particularly under-studied. Our work revealed that cellular dormancy is associated with localized regions enriched in NK cells, CD103^+^ cDCs, monocytes, and neutrophils. These regions are distinct from those surrounding proliferative tumor lesions that have abundant CD4^+^ and CD8^+^ T cells, alveolar macrophages, and DCs. *In vivo* depletion of cDC and CD8^+^ T cells established causal roles of these cells in the observed cellular tumor dormancy. The immune niche for dormant lesions is associated with IFN-γ production upon abrogation of myeloid TGF-β signaling^47–49^, a conclusion supported by a prior report^84^ and a study using scRNAseq^83^.

It was surprising given these findings that our imaging studies showed that the CD103^+^ cDC and the CD8^+^ cells involved in IFN-γ production and induction of cellular dormancy were not immediately adjacent to the quiescent tumor cells, which resided in Niche 2 with colocalized NK cells. One possible explanation for these findings is that the adaptive immune response takes time to develop after tumor introduction. The early arriving tumor cells would enter the proliferative phase, and their antigens would promote the adaptive response, but by the time the activated CD4^+^ and CD8^+^ T cells arrive at the metastatic locale, the growing tumor cells are not readily induced into the dormant state and are controlled by active anti-tumor effector function in Niche 1. Other tumor cells that are delayed in proliferation and that likely express high levels of the IFN-γR can sense the IFN-γ produced by these effector lymphocytes. Several studies measuring the dimensionality of IFN-γ action in tissues show that there are biologically meaningful effects 100–300 um from the producer cells^85–87^. This is more than sufficient to act outside of the definition we have for Niche 1 radius, maintaining the quiescent state of tumor cells capable of SLURP1-mediated dormancy induction. CD200 expression that is induced at the same time would then protect these cells from killing by the NK cells in the Niche 2 environment. Detailed studies that combine spatial transcriptomics and highly multiplex protein imaging will be needed across time series to test this hypothesis.

Our study identified SLURP induction by IFN-γ induced signaling as a key step in induction of cellular dormancy. Functional and biochemical experiments suggest that SLURP1 functions through a cell-matrix interface by interacting with fibronectin-integrin pathways to block FAK-mediated cell proliferation. This is supported by previous reports of downregulation of uPAR under these conditions that lead to reduced integrin and MAPK signaling^51^. Extracellular matrix has been reported to play an important role in both sustaining dormancy and in tumor cell emergence from this state. For example, dormant tumor cells upregulate COL17A1 or type III collagen, which contribute to maintenance of dormancy^31, 32^. Chemotherapy disrupts COL17A1 and inhibits the dormant state through FAK-YAP activation^31^. On the other hand, degrading the fibronectin matrix by matrix metalloproteinase 2 (MMP-2) promotes cancer cell outgrowth^30^. Additionally, the interaction of L1CAM with laminin in perivascular basement membranes also promotes exit from dormancy^88^. In part this is mediated through the activation of YAP and MRTF^82^. Targeting integrin αvβ6-TGFβ-SOX4 pathway by an integrin αvβ6/8-blocking monoclonal antibody sensitizes TNBC cells to cytotoxic T cells in highly metastatic murine TNBC models that were poorly responsive to PD-1 blockade^89^. A key challenge moving forward is identifying the SLURP1 interacting extracellular proteins and elucidating their functional roles in dormancy regulation.

Our studies highlight the KLF4-SLURP1-Integrin-FAK-ERK and CD200-CD200R1 axes as vulnerabilities of dormant cell states and the molecular determinants of communication with their residing niches. The strong correlation between these pathways and breast cancer patient survival and treatment relapse highlights their potential as therapeutic targets for preventing and treating metastatic diseases. We suggest that exploring targeting of CD200 could result in therapeutic translation of our findings when used in combination with chemo-and ICB. This is particularly important in addressing challenges associated with ICB and dormancy-associated relapse. Notably, in early-stage breast cancer patients treated with endocrine therapy, metastases can emerge gradually between 5 to 20 years post-diagnosis and treatment^3, 11, 90, 91^. Drugs that directly kill the dormant cells or maintain them in a dormant state will likely provide long-term benefits for breast cancer patients^25, 39, 92–94^, as supported by an NR2F1 agonist that suppresses metastasis by inducing cancer cell dormancy^26, 27^. However, the subtle interplay of immune factors in mediating both tumor cell control versus induction of dormancy that preserves viable cells able to emerge as late clinically relevant metastases suggests that harnessing this information effectively will require exceedingly careful attention to effective balancing the desired vs. the undesired effects of immune manipulation.

## Material and Methods

### Mouse strains and cell lines

*Tgfbr2* floxed (Flox cont.) mice were bred with *Lysozyme 2* promoter-driven *Cre* recombinase (LysM-Cre) mice to generate mice with myeloid-specific deletion of *Tgfbr2* (Tgfbr2^MyeKO^) as previously described^1^. Transgenic mice expressing *Tgfbr2*-shRNA were crossed with rtTA floxed mice (Strain #005670, The Jackson Laboratory) and then bred with LysM-Cre mice to generate Doxycycline (dox)-inducible myeloid-specific Tgfbr2 knock-down (Tgfbr2^MyeKD^) mice. The Tgfbr2^MyeKO^ and Tgfbr2^MyeKD^ mice genotypes were confirmed by PCR analysis of tail biopsies, using primer sequences listed in Supplementary Table 1. Female mice aged 6–8 weeks were used for animal studies. All animal procedures reported in this study were performed by NCI-CCR affiliated staff following the animal protocol LCBG007–4 and were approved by the NCI Animal Care and Use Committee (ACUC), with federal regulatory requirements and standards. AAALAC International accredits all components of the intramural NIH ACU program. All mice were maintained at a 12 h light-dark cycle, 64 – 72 °F (18 – 22 °C) temperature, and 30 – 70 % humidity condition and euthanized by following the NCI CO2 euthanasia protocol.

4T1 (#CRL-2539) cells were purchased from the American Type Culture Collection, D2.OR and D2A1 cell lines were kindly provided by Dr. Ann Chamber’s lab. The TSAE1 cell line was kindly provided by Dr. Lalage M. Wakefield of the Laboratory of Cancer Biology and Genetics, National Cancer Institute. Lenti-X 293T cells (#632180), a subclone of HEK 293 cells, were purchased from Takara Bio. All cell lines were cultured in DMEM medium (Gibco) supplemented with 10% FBS (Gibco), 100 U/ml penicillin, and 50 μg/ml streptomycin (Sigma, St Louis). All cell lines were confirmed to be mycoplasma negative by the MycoAlertTM Mycoplasma Detection Kit (Lonza) and kept in liquid nitrogen when not used.

### Mouse models of metastasis and tumor dormancy

For orthotopic metastasis studies, 4T1 cells (2×10^5^) were injected into mice’s mammary fat pad (MFP) #2. For tumor dormancy studies using experimental metastasis models, 4T1 cells (1×10^5^), D2A1 cells (2×10^5^) or TSAE1+mHer2 cells (5×10^5^) were injected through the tail vein (TVI). For survival study, 0.5–1 ×10^6^ D2A1 tumor cells were injected. The number of lung metastatic nodules was evaluated by Indian ink staining by direct counting via a dissection microscope or by lung surface imaging.

For dormancy-associated studies, when indicated, 4T1 or D2A1 cells were stained with CellVue Claret (CVC) dye (Millipore Sigma, MINICLARET) according to the manufacturer’s instructions followed by TVI. For lung surface imaging, 1mg/ml of doxycycline (Millipore Sigma, #D9891) was added to the mice water, or Cumate (Millipore Sigma, # 268402) dissolved in 70% ethanol (Sigma, # 268402) was subcutaneously injected 3 days before endpoint to induce the expression of H2B-GFP. After the animals were sacrificed, lungs were inflated with PBS, imaged using the EVOS Cell Imagining System (Thermo Fisher, M7000), and H2B-GFP+ tumor cells were individually counted. 1–8 celled H2B-GFP+ lung lesions were considered dormant lesions and lesions with greater than 8 cells were considered proliferating lesions.

### In vivo immune cell depletions and treatments

All immune cell depletion antibodies were purchased from BioXcell. For in vivo depletion, For CD8^+^ T cell depletion, mice were intraperitoneally injected with *InVivo*MAb anti-mouse CD8α (YTS 169.4, 200ug/mouse) or IgG2b isotype control (LTF-2; 200ug/mouse) antibodies every 3 days starting from day −1 of D2A1 cell injection until mice were sacrificed on day 12. For NK cell depeletion, mice were intraperitoneally injected with *InVivo*MAb anti-mouse NK1.1 (PK136, 200ug/mouse) or IgG2a isotype control (C1.18.4; 200ug/mouse) antibodies every 3 days starting from day −5, −1 and +5 of D2A1 cell injection until mice were sacrificed on day 12

For immunotherapy experiments, cancer cells were injected into mice via the tail vein on day 0, mice were then randomly grouped before various treatments. In the experiment of targeting dormant tumor cells, Tgfbr2^MyeKO^ and wt control mice were intraperitoneally treated with InVivoMAb anti-PD1 (RMP1–14, 100ug/mice) or anti-CD200 (OX-90, 200ug/mice) or in combination, with IgG2a (2A3, 200ug/mice) as control at day 3, 6, and 9. Mice were sacrificed and imaged at day 12. For the anti-CD200 effect on tumor dormancy in preclinical mouse model studies (schematic Figure 7I), mice received Cisplatin plus anti-PD1 or IgG2a as standard care on day 15, 22 (first cycle), and then on day 29, 31 (second cycle), in combination with or without anti-CD200. The mice were sacrificed and imaged on day 33 or 41 for effect on dormant tumor lesions. For mouse modeling of ICB relapse (schematic Figure 7K), the mice were treated with the same standard chemo + ICB regime as in Figure 7I till day 36, then these treatments were stopped until day 58. For anti-CD200 prevention and treatment of ICB relapse, the anti-CD200 or IgG were given on day 29, 36,41 and day 48, the mice were then sacrificed and imaged on day 58 for metastasis.

### Single-cell suspension

Lungs were collected from tumor-bearing mice, minced on ice, and transferred to gentleMACS C Tubes (Miltenyi, 130-093-237) containing RPMI (Gibco) and tumor digestion enzymes (Miltenyi, #130-093-237). Lung tissue was digested using the gentleMACS^™^ Dissociator (Miltenyi, 130-096-730) according to the manufacturer’s instructions. Following tissue digestion, red blood cells were lysed with ACK lysis buffer (Quality Biological, # 118-156-101) and cells were resuspended with RPMI supplemented with 10% FBS. The cells were washed with PBS and filtered through a 70uM cell strainer to generate a single-cell suspension for downstream analysis.

### Imaging flow cytometry

CVC labeled H2B-GFP D2A1 and 4T1 cells were injected through the tail vein, and the lungs were collected 12–14 days after injection. 3–5 million single suspended cells isolated from lung tissue were stained with 1:1000 LIVE/DEAD^™^ Fixable Near IR Dead Cell Stain Kit (Thermo Fisher, L34975) for 30 min at room temperature. Cellular non-specific binding sites were blocked with Fc blocking antibody (CD16/32 antibody, BioLegend, #101320) for 15 min. The cellular samples were then fixed, permeabilized, and stained with conjugated with P-p38-PE (Invitrogen, #12-9078-42), GAS6 (R&D systems, #AF986), and SLURP1(abbexa, #abx129448) antibodies for 40 minutes. After washing, the samples were stained with secondary antibody BV421 for Slurp1 and AF568 for GAS6 for 30 min. All the staining incubations were performed at 4 °C in dark conditions. The fluorescence intensities were measured with a 2-camera, 4-laser, 12-channel Amnis ImageStream MkII (Cytek Bioscience) imaging flow cytometer. The images were generated with INSPIRE software and further analyzed for fluorescence intensities on IDEAS 6.02 (Cytek Bioscience).

### Cell sorting

CVC labeled H2B-GFP D2A1 and 4T1 cells were injected through the tail vein and the lungs were collected 12–14 days after injection. Dissociated cells were sorted on a Beckman Coulter MoFlo Astrios EQ. Single color controls were used for spectral compensation and to establish sorting gates to collect GFP+ CVC^Pos^ and GFP+ CVC^Neg^ cells.

### Immune cell profiling with spectral flow cytometry

A multicolor antibody panel covering myeloid to lymphoid cells was used to analyze cells on the Aurora spectral flow cytometer (Cytek Bioscience). The list of antibodies is provided in Supplementary Table 2. 3–5 million single suspended cells from lung tissue were blocked with Fc blocking antibody (anti-CD16/32 antibody, BioLegend, #101320) for 30 min at 4 °C and stained with 1:1000 LIVE/DEAD^™^ Fixable Blue Dead Cell Stain Kit (Thermo Fisher, L34962). Next, cells were stained with the corresponding antibody cocktail at 1:100 dilution prepared in FACS buffer (PBS with 2% FBS and 1mM EDTA) for 30 min at 4 °C. Following 30 min cell fixation (Thermo Fisher, # 00-5523-00), cells were permeabilized and intracellularly stained with intracellular antibodies at 1:60 dilution for 40 min at room temperature. Samples were acquired on a Cytek Aurora spectral flow cytometer (Cytek Biosciences), and data were analyzed in FlowJo software v10.6.0 (BD Bioscience).

To examine effect of SLURP1 on downstream integrin signaling, D2A1 cells were cultured in Matrigel and treated with 50nM rSLURP1 (Abbexa, abx166123) or rGST (Abbexa, #abx655551) as a control for 24 hour. Cells were collected from Matrigel culture as previously described with a few modifications (PMC8202162). In brief, cells were washed with warm PBS and incubated for 30 min in chilled cell recovery solution (Corning, # 354253). Cells were then treated with TrypLE Express Enzyme (Gibco, # 12604–021) for 10 min at 37°C. Cells were fixed with fixation buffer (Invitrogen, # 00-5523-00) and washed with FACs buffer to make single cell suspension for staining.

### *Ex-vivo* co-culture of tumor cells and immune cells

Flox cont. and Tgfbr2^MyeKO^ mice were tail vein injected with D2A1 cells. 12 days later, CD8^+^ T cells and CD103^+^ DCs were sorted from the lungs. Immune cells were cultured with CVC-labeled H2B-GFP-D2A1 cells in GlutaMAX RPMI 1640 medium (Gilbco, #61870036), supplemented with 10% FBS, 1000 IU/ml murine recombinant IL-2 (Peprotech, #212–12), 1ug/mL anti-CD3 (eBioscience, # 16-0031-82), and 1ug/mL anti-CD28 (eBioscience, #14-0281-82) in the presence or absence of IFN-γ (BioXcell, BE00550) or TNF-α (BioXcell, BE0058) neutralization antibodies for 3–5 days. 24 hours before Ki-67 and Cleaved caspase3 staining, cells were treated with 2μg/ml dox to induce H2B-GFP expression in D2A1 cells. The ratio of D2A1 cells to CD103^+^ cDCs (1:1) and CD8+ T cells (1:2) was used.

To examine the effect of CD200 cancer cell expression on NK cell and macrophage cytotoxicity, flox cont. and Tgfbr2^MyeKO^ mice were tail vein injected with D2A1 cells. NK cells and macrophages were sorted from CD200R1^+^ splenocytes 12 days later and cultured overnight in RPMI 1640 (Gibco) with 10% FBS (Gibco), 0.375% sodium bicarbonate, 1mM sodium pyruvate, 0.0001% 2-mercaptoethanol and 1000 IU/ml murine recombinant IL-2. Control or CD200 overexpression D2A1 cells were then added to immune cell cultures for 24 hours before measuring cytotoxicity using the CytoTox96 Cytotoxicity Assay (Promega, # G1780). The ratio of D2A1 cells to NK cells and macrophages was 1:1. Percent cytotoxicity was calculated using a maximum LDH release control and the formula: $\text{Percent cytotoxicity}=100\times \frac{\text{Experimental LDH Release}\;\left(OD490\right)}{\text{Maximum LDH Release}\;\left(OD490\right)}$ as described via manufacturer instructions.

### Mouse tissue immunofluorescence staining using iterative bleaching and extended multiplexity (IBEX) and analysis

Lung tissues were prepared, imaged, and aligned as previously described with minor modifications (https://www.biorxiv.org/content/10.1101/2024.07.04.601620v1). In brief, lungs were inflated with Cytofix (BD) and kept in BD Cytofix/Cytoperm (BD BioScience) at 4 °C for 14–16h. The next day, tissues were washed with PBS once, incubated in 30% sucrose for 24h at 4 °C, embedded in OCT compound (Tissue-Tek), and kept at −80°C until use. Frozen lung tissues were cut at 20–30 μm on a CM3050S cryostat (Leica) and adhered to Superfrost Plus slides (VWR, Cat. # 48311–703) coated with 15 μL of chrome alum-gelatin adhesive (Newcomer Supply, Cat. #1033A). 20–30 μm lung tissue serial sections were adhered to chrome alum gelatin (Newcomer Supply) coated slides and kept overnight to dry. Tissue sections were permeabilized in BD perm buffer supplemented with mouse Fc block (BD) and Hoechst 33342 (Biotinum). Serial sections were screened with a Leica THUNDER imaging system equipped with a 20× objective (NA 0.75) (Leica, Cat #11506343), and dormant tumor cells/colonies (less than 8 cells) were identified based on GFP signals and used for subsequent IBEX procedures. Tissues were stained with PELCO BioWave Pro-36500-230 microwave (Ted Pella) for staining. Two cycles of 2-1-2-1-2-1-2-1-2 program were used for immunolabeling with the BioWave, where “2” denotes 2 min at 100 W and “1” denotes 1 min at 0 W. After staining, slides were mounted with Fluormount G (Southern Biotech, Cat. #0100-01) and examined on a Leica THUNDER Imaging system or STELLARIS confocal microscope. Fluorochrome inactivation was performed with 1 mg/mL of LiBH_4_ (STREM Chemicals) for 15 minutes. Samples were further stained with the antibodies for the subsequent cycle of imaging. SimpleITK program was used for image alignment, followed by downstream analysis. Confocal images were collected using a Leica Stellaris 8 laser scanning microscope equipped with an HC plan-apochromat 20x (N.A. 0.75) multi-immersion objective lens with 0.284 μm x-y pixel size and 4.0 um optical section thickness. The antibodies were provided in Supplementary Table 3.

### IBEX analysis

#### Preparing Multiplex Mouse Lung Images for Analysis

Each channel of the mouse lung multiplex images was restricted to its dynamic range of expression by saturating the brightest 0.01% of pixels. Images were then converted to 8-bit format. Noise due to autofluorescence and channel spillover was evident by its similar appearance across multiple channels, and it was reduced via pairwise channel arithmetic.

The Hoechst nuclear stain was binarized to segment individual cells, followed by a watershed step to separate touching nuclei. Raw signal for every membrane marker (all markers except Ki67, GFP, and αGFP) was passed through a Gaussian filter with a four-pixel radius to increase the overlap of membrane stain with the nucleus of the expressing cell. The size, centroid location, and mean fluorescence intensity (MFI) of each marker was then measured for each cell in each image. All steps were performed in ImageJ.

### Classifying Cells into Phenotypes

Using the single-cell MFIs for each marker, a threshold was drawn at the “elbow” of the expression distribution for each marker to separate positive from negative expression. Each cell’s profile of +/− expression for each marker was compared to expected expression profiles for known cell types, and cells that matched an expected type were categorized as such. The MFIs for these cells were then used to define representative quantitative expression profiles for each known cell type.

The quantitative expression profile for each remaining uncategorized cell was then used as the output variable in multivariate linear regression, with the representative quantitative expression profiles for each known cell type as multivariate predictors. Essentially, this approach models each uncategorized cell as a mixture of the representative expression profiles for known types. Among known types that contribute significantly to an uncategorized cell’s expression profile, that with the largest contribution was selected as the uncategorized cell’s type.

This left 7.7% of cells that were not significantly associated with a known type. These were subject to unsupervised k-means clustering to search for unexpected cell types. However, the clusters that emerged were typically characterized by the expression of just a single marker, which was visually confirmed to correspond to leftover noise in the images. Thus, uncategorized cells were excluded from the analysis. Furthermore, the 0.4% of cells categorized as γδ T cells were also excluded from analysis due to the remaining spillover of CD11b into the γδTCR channel, which made the distinction of true γδ T cells challenging. All steps were performed in R.

### Definition of Tumor Lesions

The table of XY centroid coordinates for each cell was used to measure each tumor cell’s distance from every other tumor cell, within each image. These distances defined a graph representation of the tumor cells, in which tumor cells were considered neighbors if their distance was ≤ 200 μm. Connected components on this graph then defined individual lesions.

After the tumor cells were partitioned into separate lesions, the table of XY centroids was again used to measure the distance of each non-tumor cell to each tumor cell. All non-tumor cells within 200 μm of a tumor cell were assigned to that tumor cell’s lesion. Thus, while each tumor cell was assigned to exactly one lesion, non-tumor cells could be assigned to zero, one, or more than one lesion. All steps were performed in R.

### Analyses of Whole Lungs and Tumor Lesions

The total fraction of tumor cells in Tgfrb2^MyeKo^ vs. flox cont. lungs was compared using a binomial linear regression model. The output variable was each cell’s binary status as a tumor cell or not, and the predictors were the fixed effect of genotype and the random effect of sample ID. The size of lesions in Tgfrb2^MyeKo^ vs. flox cont. lungs were compared using a zero-truncated negative binomial model. The output variable was each lesion’s size (number of tumor cells), and the predictors were the fixed effect of genotype and the random effect of sample ID. Both models were built using the glmmTMB R package.

To explore the immune cells in each lesion, the counts of each immune cell type in each lesion were re-expressed as center-log ratios (CLRs) to account for the compositional nature of this data. The CLRs for each cell type were then standardized separately to account for the differences in total abundance across cell types. These standardized CLRs were used in place of raw cell counts for all ensuing analyses (except direct ratios of CD8 and CD4 T cells).

The pairwise correlations across cell types were measured, and the modules of co-occurring cell types were defined using the gplots R package. Principal component analysis (PCA) was also performed on the standardized CLRs in R. The significance of associations between PC1 and the size and genotype of each lesion was investigated using a Gaussian linear regression model. The output variable was each lesion’s PC1 score, and the predictors were the fixed effects of lesion size, genotype, and their interaction, and the random effect of sample ID.

### Clearing enhanced 3D imaging (Ce3D)

Volumetric imaging with optically cleared samples with Ce3D was performed as described previously with slight modification (https://www.pnas.org/doi/10.1073/pnas.1708981114) (https://www.biorxiv.org/content/10.1101/2024.07.04.601620v1). Briefly, frozen lung samples were sectioned at 300 μm on a CM3050S cryostat (Leica Biosystems). The samples were hydrated and washed with PBS to remove OCT in a 24-well plate. Samples were incubated for at least 12 hours in BD Perm/Wash Buffer (BD Bioscience) containing 1% mouse Fc block (BD Bioscience, Cat. #553142) and stained with titrated antibodies in BD Perm/Wash Buffer (BD Bioscience, Cat. #554723) for 24 hours at room temperature on a shaker. Stained samples were washed with BD Perm/Wash Buffer three times for at least 20 minutes at room temperature on a shaker and transferred on a slide with two silicon isolators (Grace BioLabs, Cat. #664407) and treated with 200 μL of Ce3D medium [1.82 g Histodenz (Millipore Sigma, Cat. #D2158–100G, 0.1% triton, and 0.5% thioglycerol (Millipore Sigma, Cat. #M1753) per 1 mL 40% N-methylacetamide (Millipore Sigma, Cat. #M26305–500G) in PBS] inside a chemical fume hood and sealed with a cover slip (Electron Microscopy Sciences, Cat. #63766–01). Samples were protected from light and incubated at room temperature on a shaker overnight. Cleared samples were mounted with 40 μL of new Ce3D and sealed with a coverslip with two SecureSeal Imaging Spacers (Grace Bio-Labs, Cat. #654002) and examined on a Leica STELLARIS confocal microscope equipped with a 20 × objective (NA 0.75).

### Sphere formation assay

Matrigel (Corning, #356231) was added to 96-well plates or 15 μm-slide (ibidi, #81506) on ice and solidified by incubating at 37°C incubator for 40 min. Tumor cells were suspended in culture media with 2% Matrigel. 500 cells/96 wells were added with varying concentrations of rSLURP1 (Abbexa, abx166123) or rGST (Abbexa, #abx655551) as a control. Cells were cultured for 3–5 days and supplemented with fresh recombinant protein every 24 h. For Integrin activation, 1000 D2A1 cells were seeded in Matrigel-coated 96-wells. Cells were then pretreated for 1 h with 500 μg/mL integrin activation antibodies or IgG (BioXcell, BE0232, or BE0089) before adding 50nM of rSLURP1. Cells were supplemented with fresh recombinant protein every 24 h and cultured for 5 d before imaging. For rSLURP1 withdrawal, D2A1 cells in Matrigel-coated 96-wells were treated with 50nM rSLURP1 for 5 days. On day 5, cells were washed with 0% FBS media to remove recombinant protein, supplemented with fresh 10% FBS media, and allowed to grow for 3 more days before imaging. Relative proliferation and spheroid area were measured using ImageJ software (https://imagej.net/ij/).

### RNA-seq of sorted dormant, proliferative tumor cells, tumor cells with rSLURP1 treatment

GFP+ CVC^Pos^ (dormant) or GFP+ CVC^Neg^ (proliferating) D2A1 cells were sorted from the lungs of WT or Tgfbr2^MyeKO^ mice 12 days after TVI. Cells were sorted into lysis buffer and an aliquot of 50 cells (determined by Flow Sorter). For 3D spheroid cultured cells. rGST or rSLURP1 treated cells were harvested as described (PMC8202162) on day 5. Samples were subjected to Reverse Transcription using QIAGEN RNeasy Kit (#74104) followed by library preparation using Smart seq2 protocol (Nature Protocols, 9:171–181, (2014).

The sequencing reads were processed for adapter trimming using Cutadapt (version 1.14) to remove unwanted adapter sequences and low-quality bases at the read termini. The sequencing quality of the trimmed reads was assessed per sample using FastQC (version 0.11.5) to evaluate base quality scores, adapter contamination, and sequence duplication levels. Besides FastQC, Preseq (version 2.0.3) was used to estimate library complexity and predict the yield of unique reads for deeper sequencing. Picard tools (version 1.119) were employed to assess metrics, such as insert size distribution, duplication rates, and sequence alignment summaries. To further ensure data quality, RSeQC (version 2.6.4) provided insights into RNA integrity, GC content, and potential biases in sequencing coverage.

The trimmed reads were then aligned to the mm10 mouse genome using STAR (version 2.5.2b) in two-pass mode to enhance mapping sensitivity and accuracy. After successful alignment, gene expression levels were quantified using RSEM (version 1.3.0), which employs an expectation-maximization algorithm to accurately estimate gene and transcript abundance. The reference genome annotation used for expression quantification was GENCODE annotation M12.

Gene-level expression estimates from RSEM were used in downstream DESeq2 analysis to identify differentially expressed genes between the conditions shown in Fig 2B. The statistical significance of differentially expressed genes was determined using a q-value threshold of ≤0.05 and an absolute fold change cutoff of ≥ 2. Each of these comparisons aimed to elucidate the transcriptional differences between dormant and proliferative states, as well as the impact of Tgfbr2 deletion. Further downstream analyses, including hierarchical clustering and principal component analysis (PCA), were performed to visualize sample relationships and validate the robustness of expression changes. To gain insights into the functional relevance of differentially expressed genes, pre-ranked Gene Set Enrichment Analysis (GSEA) was performed to assess pathway-level enrichment.

Immune evasion genes enriched in dormant tumor cells from Tgfbr2MyeKOmice were identified through pathway analysis using QIAGEN Ingenuity Pathway Analysis (IPA). Differentially expressed input genes were defined using the q-value threshold of ≤ 0.05 and an absolute fold-change cutoff of ≥ 1.5. Data is presented as TPM values of each gene, with 3 independent variables.

### Lentivirus production and transduction

The predesigned shRNA plasmids were purchased from Millipore Sigma. The catalog numbers and sequences of shRNA used are provided in Supplementary Table 4. The mRuby-p27K^−^ quiescence reporter was kindly provided by Dr. Steven Cappell from the Laboratory of Cancer Biology and Genetics, National Cancer Institute. The packaging plasmid psPAX2 (#12260) and the PMG2.G (#12259) envelope plasmid were obtained from Addgene. The mouse coding sequences for Slurp1, Klf4, and CD200 were cloned into the FerH-NeoR-Lenti overexpression vector and sourced from GenScript Biotech. For lentivirus production, 80% confluent LentiX cells were transfected with lentiviral vector, psPAX2, and PMD2.G by Lipofectamine 3000 reagent (Invitrogen, # L3000001) in OptiMem medium. The medium was replaced with fresh media 4–6 h after transfection. After 48 h, viral supernatant was collected, centrifuged, and filtered through 0.45 μm filters and stored at −80°C. For lentivirus transduction, indicated cells were incubated with viral supernatants and 8 ug/mL Polybrene for 24 h. Successfully transduced cells were selected by adding the appropriate antibiotic into the culture medium 48 −72 h after transduction.

### Slurp1 sgRNA-induced gene knockout

D2A1 cells were transduced with Pspcas9-2A-puro-px459 (Addgene 48139) constructs containing Slurp1 single-guide RNAs designed by F. Zhang’s laboratory, which was followed by 2 μg/ml puromycin selection for 2 d. Single clones were collected and knockout was confirmed by genotyping PCR fragments consisting of the target sequences. Slurp1 sgRNAs and PCR validation primer sequences are listed in Supplementary Table 5.

### RT-qPCR

RNA was isolated using the Quick-DNA/RNA Miniprep Kit (Zymo Research, # D7001), and 1μg of cDNA was synthesized using the ReverTra Ace qPCR RT Kit & Master Mix (Toyobo, # FSQ-101). RT-qPCR reactions were carried out using the PowerSYBR Green PCR Master Mix (Thermo Fisher, # 436765) on a QuantStudio 6 Flex Real-Time PCR System (Thermo Fisher, #4485691). For SLURP1 induction, D2A1 and 4T1 cells were serum starved overnight before treatment with recombinant murine IFN-γ (10ng/mL) (Cell Signaling, #39127S) for 24 hours. mRNA levels were normalized to the housekeeping gene Gapdh, and relative fold change was determined using the 2−ΔΔCt method. All primer sequences are provided in Supplementary Table 6.

### Western blotting

Cells were lysed with RIPA Lysis Buffer (Sigma, #R0278–50ML) supplemented with cOmplete protease inhibitor cocktail (Roche, #04693132001) and phosphatase inhibitor cocktail (Roche, #4906845001). Protein concentration was measured by Pierce’s BCA Protein Assay (Thermo Fisher, #23225). An equal amount of total protein (20–40μg) was denatured, separated on NuPAGE 4–12% Bis-Tris protein gels (Thermo Fisher, #NP0321), and transferred to a nitrocellulose membrane (Bio-Rad, #1704270). Membranes were blocked in 5% non-fat milk in TBS and .1% Tween 20 (TBS-T) for 1 hour at room-temperature before overnight incubation in primary antibodies against SLURP1 (Provided by Yasuhiro Moriwaki, Keio University), IFNGR1 (Cell Signaling, #84318S), pSTAT1 (Cell Signaling, #9167S), STAT1 (Cell Signaling, #9172S), KLF4 (abcam, #ab129473), GAPDH (Cell Signaling, #2118) or β-actin (Santa Cruz, sc-69879, AC-15) at 4 degree. Primary antibodies were diluted 1:1000. Secondary HRP-conjugated anti-mouse (Cell Signaling, #7076S) and anti-rabbit (Cell Signaling, #7074S) were used at a 1:2000 dilution. Protein detection was done using the ECL reagent (Bio-Rad, #1705061) and visualized using the ChemiDoc imaging system (BioRad).

### BioPlex Immunoassay

Flox cont. and Tgfbr2^MyeKO^ mice were tail vein injected with 2×10^5^ D2A1 cells, and lungs were collected after 12 days. Lungs were flash-frozen and stored in −80 until use. Lung tissue was homogenized with the Precellys Lysing Kit (KT03961-1-002.2, Precellys) using the Precellys 24 tissue homogenizer (P002391-P24T0-A.0, Bertin Instrument). Protein concentration of cytokines was measured and quantified using the Bio-Plex Pro Mouse Cytokine 23-plex Assay according to the manufacturer’s protocol (Biorad, M60009RDPD).

### KLF4 ChIP-Slurp1 RT-qPCR

The ChIP assay was performed following the protocol provided by Active Motif (catalog #53008). Briefly, 1 × 10^7^ KLF4 OE and control cells were treated with 1% formaldehyde for 10 min at 37°C to cross-link proteins with DNA. Cells were then lysed in SDS buffer for 10 min on ice, followed by sonication (30% of maximum power, 15 s on, 1 min rest, for a total of 25 min) to generate DNA fragments ranging from 200 to 1000 bp. The samples were precleared with protein A beads for 30 min on a rotor at 4°C and then incubated overnight at 4°C with 2 μg anti-KLF4 or control antibodies. After a series of washes, the eluted fractions were subjected to DNA isolation and analyzed by qPCR. Primer sequences used to map Slurp1 promoter regions are listed in Supplementary Table 7.

### DNase I sensitivity assay

For the DNase I sensitivity assay, KLF4 OE and control cells were collected, and nuclei were isolated following the protocol of the nuclei isolation kit (catalog #78833). The nuclei pellets were resuspended in DNase digestion buffer, and 0 or 2 U of DNase I (EN0521) was added, followed by a 5 min incubation at 37°C. After terminating the digestion, genomic DNA was extracted, and the indicated chromatin regions were analyzed by qPCR.

### Human correlative studies

Publicly available datasets from breast cancer patients (METABRIC and TCGA) were analyzed to determine *SLUPR1* expression in different subtypes. For survival analysis, patient survivals were correlated with *SLUPR1* expression or a two-gene signature combining *SLURP1* expression with negatively weighed *TGFBR2* expression in the METABRIC, TCGA and Kaplan-Meier Plotter datasets. For cell type-specific gene expression analysis, the computationally deconvolved TCGA-BRCA dataset by CODEFACS (https://zenodo.org/record/5790343) was utilized (ref). To define a cell type-specific gene signature, *SLURP1* expression from cancer cells was integrated with negatively weighed *TGFBR2* expression from monocytes and neutrophils. The correlation between CD200 expression in cancer cells and CD200R1 expression in immune cells were assessed using publicly available human breast cancer scRNA-seq data (Nature Medicine, 2021, https://lambrechtslab.sites.vib.be/en/single-cell). The correlation between IFNγ and dormancy gene signature, including SLURP1, IFNGR1, IFNGR1, CD200 expression in cancer cells and CD200R1 expression in NK and T cells, was investigated using publicly available scRNA-seq data of human breast cancer (Cancer Cell, 2024, https://www.ncbi.nlm.nih.gov/geo/query/acc.cgi?acc=GSE246613).

### Statistics & Reproducibility

All data are presented as mean ± Standard Error of the Mean (s.e.m.). Unless otherwise indicated, comparisons between two groups were performed using a two-tailed Student’s t-test. Statistical analysis of survival data used the Wilcoxon test. Statistical analyses were performed using GraphPad Prism 7.0 software, and significance was defined as *P* <0.05. Significance is noted in figure or figure legends; *P*-value as *P* < 0.05, *P* < 0.01, *P* < 0.001, *P* < 0.0001, NS > 0.05. No data were excluded from the analyses, except for Supplementary Figure 1A, in which one replicate was excluded due to an outlier. All mice used in *in vivo* studies were randomized before cancer cell injection. The phenotype of animal experiments was not evaluated blindly, and multiple researchers verified major results.

## Supplementary Material

Supplementary Files

This is a list of supplementary files associated with this preprint. Click to download.

- supplefigures11222025bprintLY.pdf

## Data Availability

The data supporting the findings of this study are available within the article, and the supplementary information files are available from the corresponding author upon request. The raw and processed RNA-sequencing data have been deposited in the GEO database and are publicly available under accession numbers (GSE292496) and (GSE292514). All relevant source data for each figure are provided.

## Significance

Our unexpected discovery that the IFN-γ rich immune microenvironment, paradoxically induces tumor dormancy, demonstrate a yet to be recognized duality to the effects of immune check point blockade (ICB). In addition, the identification of KLF4-CD200-CD20R in immune niche formation and immune evasion of dormant tumor lesions provide targeting opportunities for ICB resistance and relapse.
